# Challenges With the Development of Biomaterials for Sustainable Tissue Engineering

**DOI:** 10.3389/fbioe.2019.00127

**Published:** 2019-05-31

**Authors:** David F. Williams

**Affiliations:** ^1^Wake Forest Institute of Regenerative Medicine, Winston-Salem, NC, United States; ^2^Strait Access Technologies, Cape Town, South Africa

**Keywords:** template, biocompatibility, scaffold, biomaterial, biodegradation

## Abstract

The field of tissue engineering has tantalizingly offered the possibility of regenerating new tissue in order to treat a multitude of diseases and conditions within the human body. Nevertheless, in spite of significant progress with *in vitro* and small animal studies, progress toward realizing the clinical and commercial endpoints has been slow and many would argue that ultimate goals, especially in treating those conditions which, as yet, do not have acceptable conventional therapies, may never be reached because of flawed scientific rationale. In other words, sustainable tissue engineering may not be achievable with current approaches. One of the major factors here is the choice of biomaterial that is intended, through its use as a “scaffold,” to guide the regeneration process. For many years, effective specifications for these biomaterials have not been well-articulated, and the requirements for biodegradability and prior FDA approval for use in medical devices, have dominated material selection processes. This essay argues that these considerations are not only wrong in principle but counter-productive in practice. Materials, such as many synthetic bioabsorbable polymers, which are designed to have no biological activity that could stimulate target cells to express new and appropriate tissue, will not be effective. It is argued here that a traditional ‘scaffold’ represents the wrong approach, and that tissue-engineering templates that are designed to replicate the niche, or microenvironment, of these target cells are much more likely to succeed.

## Introduction

This paper, and this journal issue in general, is concerned with sustainability in the field of tissue engineering. It is an opportune time to reflect on this topic; sustainability refers either to a position that is demonstrably correct and defendable or an activity that can be continued and developed within the foreseeable future. In reality these meanings are linked since it should not be possible to successfully continue an activity, especially a complex activity such as tissue engineering, without it being correct and defensible. We should, therefore, examine the sustainability of tissue engineering, and, in particular, the role that biomaterials play in this.

In order to make it absolutely clear what sustainable tissue engineering means here, a few definitions and concepts need to be addressed. Tissue engineering is the creation of new tissue for the therapeutic reconstruction of the human body, by the deliberate and controlled stimulation of selected target cells through a systematic combination of molecular and mechanical signals (Williams, 2006). While this does not directly imply that tissue engineering has to involve biomaterials, the delivery of those molecular and mechanical signals cannot take place in a vacuum, and there will usually have to be a vehicle that accurately controls the relevant processes. Such vehicles have usually been described as scaffolds. However, the term “template” is preferred as this involves a different concept and avoids the old-fashioned ideas of scaffold biomaterials. Tissue engineering may be considered as one of the scientific platforms of regenerative medicine, the others being cell therapies and gene therapies.

As discussed by Vacanti back in 2006 (Vacanti, 2006), tissue engineering has its origins in the late 1980s; in other words, we now have ~30 years-worth of developments in this field. Many people, including myself, have written about the “promise” of tissue engineering, albeit with commentaries on the difficulties of achieving success. In 2004, I reviewed the benefits and risks of tissue engineering (Williams, 2004) referring to the uncertainty that exists with respect to its commercial and clinical exploitation. I noted that, during the previous decade, a number of companies had been formed with the objective of commercializing tissue engineering and that investment looked attractive; however, the tide had turned, with a number of high-profile bankruptcies and changes in company positions, with little hope of recovering initial investments. Two fundamental issues were at stake; on the one hand, the R & D costs were very high and on the other hand there was little prospect of these companies being able to sell their products for a reasonable sum, since the investigational nature of the work meant that treatment was not reimbursable under most insurance schemes.

I returned to this theme over 10 years later (Williams, 2017a) stating that the field had still not yet resulted in the widespread clinical and commercial successes that were initially predicted; there were also some problem areas with the conduct of clinical research, which had set the field back. There are many reasons why tissue engineering has not yet fulfilled the initial promises, and many of these, as alluded to above, are concerned with the infrastructure, including those related to health economics and reimbursement, regulation, ethics and bioprocessing. However, if the scientific principles upon which these promises rested had been fully developed, ways forward or around these logistical hurdles would almost certainly have been found. The scientific factors largely relate to the ability to deliver those molecular and mechanical signals mentioned earlier, such that new tissue, with appropriate morphological and functional characteristics, can be generated and maintained. Within this complex milieu, the roles of, and effects on, the target cells, on the whole array of biomolecules and on the biomaterial templates all have to be considered. In order to determine how these templates have performed, and have contributed, either positively or negatively, to tissue regeneration, it is necessary to consider the clinical outcomes that have emerged.

## The Clinical Performance of Tissue Engineering Biomaterials and Templates

### Skin Tissue Engineering

With hindsight, the major problems with tissue engineering biomaterials were apparent from the early experiences with skin tissue engineering products and processes. The very public failure of Advanced Tissue Sciences Inc., and the difficulties with commercialization of its flagship product Dermagraft®, serves as a good example (Pangarkar et al., 2010). All of the infrastructure issues mentioned above were certainly involved, and the company was undoubtedly unlucky with respect to the reluctance of the FDA to approve new products following controversies such as the silicone breast implant scenario and the general economic situation, but it should not be overlooked that the product contained not only neonatal allogeneic fibroblasts but also a poly(lactic acid)-poly(glycolic acid) synthetic material.

From biomaterials and bioengineering perspectives, it was already becoming clear by the early 2000s that such synthetic polymers were unlikely to provide optimal substrates for tissue engineered skin. Metcalfe and Ferguson reviewed the relevant issues in 2007 (Metcalfe and Ferguson, 2007), indicating that no then-current bioengineered skin completely replicated the anatomy, physiology, biological stability or aesthetic nature of uninjured skin, with significant problems of under-vascularization, excessive scarring, a lack of complexity of differentiated structures and poor biocompatibility of the supporting membranes. These issues were stressed by van der Veen et al. (2010), who emphasized that fibroblasts need to be offered binding locations and chemotactic signals that can guide cell function and that the foreign body response to the degrading synthetic material will inhibit the wound healing process. Similar positions have been taken by Kamel et al. (2013) and Groeber et al. (2011), and in 2017, Vig et al. referred to continuing unavailability of an engineered skin substitute, with the properties of the biomaterial substrate being a major factor (Vig et al., 2017). Some indication of the better success that could be achieved with alternative substrates to the synthetic biodegradable polymers has been seen with products such as Apligraf (Stone et al., 2017); this is a skin substitute consisting of human foreskin-derived neonatal fibroblasts in a bovine Type I collagen matrix and a further layer of human foreskin-derived neonatal epidermal keratinocytes. This has been used in the treatment of venous leg ulcers, where it appears to induce a shift from a non-healing to a healing tissue response, with a modulation of inflammatory and growth factor signaling, keratinocyte activation and attenuation of Wnt/ß-catenin signaling. Clearly, the collagen matrix is far more capable of inducing such responses than synthetic polymers. This point was recently underlined by Ter Horst et al. (2018) when reviewing keratinocyte delivery in burn wound care, noting that natural biopolymer hydrogels, such as those of chitosan, alginate, fibrin, and collagen can act as supportive matrices in cell delivery, but not those of synthetic polymers.

### Articular Cartilage Tissue Engineering

Articular cartilage received similar attention to skin in the early days of tissue engineering and the transition from R. & D. to clinical and commercial applications has followed a somewhat parallel trajectory. As recently reviewed by Huang et al. (2016), there are several clinical techniques available for the treatment of focal lesions in articular cartilage, including microfracture and autologous chondrocyte implantation, but in view of the limited capacity of the avascular, low cellular, cartilage to heal, these techniques do not consistently produce hyaline repair tissue. A number of cell-based tissue engineering products have been designed over the last two decades, some of which have been subject to clinical trials. The tissue engineered cartilage constructs involved here are generally formed by the integration of chondrocytes, signals, and scaffolds. These scaffolds are, strictly speaking, of exogenous origin, although, confusingly “scaffold-free” techniques have been introduced, in which an endogenously-generated “scaffold” that is generated by cells is involved. At the present time, very few of these constructs employ a synthetic polymer scaffold. Bioseed®-C and INSTRUCT® contain either polyglactin 910/polydioxanone or poly(ethylene oxide terephthalate)/poly(butylene terephthalate) biodegradable polymers. Development of these products has been slow, with mixed results.

More products have used natural biopolymers as scaffolds. Biocart ™ II uses autologous chondrocytes within freeze-dried fibrin and hyaluronan, Cartipatchç uses an agarose-alginate hydrogel, Hyalograft®-C a non-woven mesh of hyaluronic acid-based microfibres, NeoCart® a bovine type I collagen and so on. One good example here is the Novocart® product, a 3D autologous chondrocyte implant system composed of *ex vivo* expanded autologous chondrocytes seeded on a bioresorbable biphasic collagen scaffold, which is in Phase III trials. Overall, the pre-clinical results seem better with the natural biopolymer constructs or scaffold-free approaches than with the synthetic polymers, and more of these products have entered Phase II or III clinical trials, but the huge time delay from study inception to reimbursable clinical usage is still inconsistent with a concept of sustainable tissue engineering biomaterials. A very recently published systematic review of clinical evidence in the area of therapies for focal chondral defects (Gao et al., 2019) suggests that the general difficulty of this tissue repair has resulted in a significant lack of high-quality randomized controlled clinical studies on techniques such as microfracture and autologous matrix induced chondrogenesis; few recommendations on optimal methods can therefore be made.

With biodegradable polymers such as PLGA it is interesting to note their history of pre-clinical evaluation. Just as with polymer-based skin tissue engineering products, there was initial enthusiasm about the possibility of chondrogenesis and cartilage repair within porous scaffolds seeded with chondrocytes. However, two problems emerged with such systems, concerned first with the ability of either chondrocytes or stem cells, to attach to, and function on, these polymer surfaces and secondly with the effects of polymer degradation on tissue regeneration. For example, Zanatta et al. showed that adherence of mesenchymal stem cells to a PLGA copolymer scaffold is heavily dependent on the presence of integrin- ß1 receptors (Zanatta et al., 2012), while Asawa et al. showed that inflammatory cells associated with the response to degrading PLGA scaffolds significantly affected proteoglycan and type II collagen production during autologous transplantation of tissue engineered cartilage (Asawa et al., 2012).

During the last decade or so there have been many attempts to ‘modify’ these synthetic scaffolds and the literature is replete with *in vitro* and small-animal studies on cell responses to morphological and biochemical variants of degradable polymers, especially the aliphatic polyesters. It is certainly true that nanofibrous architectures can have some influence (Xin et al., 2007), as can pore size and orientation (Zhang et al., 2012), but these rarely provide improvements of clinical relevance since they do not substantially address the main underlying problems. This does not mean that degradable polyester systems do not have any role in cartilage tissue engineering, but it would appear that their role is most likely to be as carriers (or microcarriers) of biomolecules or genes rather than as structural scaffolds or templates. Tan et al. (2009) were among the first to use PLGA microspheres to modify the properties of natural biopolymer scaffolds (gelatin/chitosan/hyaluronan). This was taken to a new level by Morille et al. (2016) who incorporated TGF ß3 into PLGA microspheres that were suspended in mesenchymal stem cell preparations. In a murine arthritic knee joint model, these encouraged chondrogenesis and the formation of cartilage-like tissue. Im et al. used a similar approach by impregnating a PLGA scaffold with plasmid DNA containing a SOX-5, -6, and -9 genes, which induced chondrogenesis of adipose stem cells (Im et al., 2011).

Bearing in mind the far greater complexity of regulatory approval and reimbursement procedures for products that contain active biomolecules and/or genes, such developments are highly unlikely to enhance the sustainability of tissue engineering biomaterials.

### Tracheal/Airway Tissue Engineering

A paper published in 2017 asked a question in its title “Tissue engineering of the trachea: what is the hold-up?” (Siddiqi, 2017). This was a sensible question, but with a complex answer. As an editorial in The Lancet in 2018 (Editorial, 2018) revealed, intensive investigations by The Karolinska Institute in Sweden and by University College, London had confirmed that experimental and clinical work by Macchiarini and colleagues on tracheal tissue engineering constituted scientific fraud and unethical behavior. This resulted in the death of several patients and the retraction of some, apparently pivotal, studies and reviews (Jungebluth et al., 2011; Badylak et al., 2012). This called into question the validity of tracheobronchial transplantation using tissue engineering techniques, and the translation of these concepts to the arena of engineered whole organs and complex tissues. There were many factors that lead to the deaths, but the scaffolds used in the procedures were clearly inadequate. Several constructs were employed, including cadaveric tracheas and a biodegradable synthetic composite material described as “POSS-PCU”, polyhedral oligomeric silsesquioxanes and poly (carbonate-urea)-urethane. It is difficult to know why the latter material failed since there were both intrinsic problems with its biocompatibility and non-compliant manufacturing issues, but this disastrous approach did not help at all with the development of sustainable tracheal tissue engineering. In reviewing the possibility of clinical translation of tissue engineered tracheal grafts, Chiang et al. (2016) referred to the inappropriate nature of this material in terms of “.*the stiffness of the material led to compliance mismatch, granulation tissue formation, the development of fistula at distal anastomotic sites, poor vascularization and epithelialization and susceptibility to infection.”* In other words, pretty well everything went wrong from a biocompatibility perspective.

Concerning the alternative scaffolds, Chiang et al. (2016) discussed decellularized tracheas, which are intended to work through removal of cellular and immunogenic material from the ECM of donated tracheas, ostensibly to preserve the mechanical and bio-inductive properties of the tissue. While conceding that variations in decellularization protocols results in significant differences in outcomes, the overall impression was that the rate of chondrocyte repopulation does not usually match the rate of ECM degradation. This point was amplified by Maughan et al. in a 2017 review of autologous cell seeding in tracheal tissue engineering (Maughan et al., 2017), for although considerable remodeling of the ECM can occur, some critical ECM signaling molecules may be removed during decellularization, while the biomechanical properties may be unfavorably modified and the resulting material may still elicit adverse immunological responses. These latter complications are consistent with current theories of biocompatibility described in detail by the present author recently (Williams, 2017b).

The current situation with tracheal tissue engineering was critically discussed by Elliott et al. (2017) (the same team that produced the review cited as 28 above), who presented the results of a case where a stem-cell seeded decellularized tissue-engineered tracheal graft was used on a compassionate basis for a girl with critical tracheal stenosis. In spite of following full GMP procedures and appropriate clinical techniques, the patient died 3 weeks post-transplantation following an intrathoracic bleed and sudden airway obstruction (Elliott et al., 2017).

While making it clear here that, in the very difficult clinical area of tissue-engineered approaches to airway reconstruction, biological materials such as decellularized tissues are far from ideal and synthetic biodegradable polymers appear to have very little chance of success, there are other biomaterials-based options. Hollister and colleagues published an experimental model of treating tracheomalacia using 3D-printed bioresorbable airway splints in 2013 (Zopf et al., 2014); this approach was used clinically in compassionate cases of tracheobronchomalacia over the next few years (Zopf et al., 2013; Morrison et al., 2015). The splint, referred to as a personalized medical device rather than a scaffold is polycaprolactone homopolymer. This is biodegradable, its supportive presence allowing natural cartilage remodeling to take place over a matter of months. This technique is still with very limited clinical application, but may provide options for the very difficult management of tracheobronchomalacia (Svetanoff and Jennings, 2018).

One implication of the latter approach, which rarely if ever is described as “tissue engineering,” is that ambitions for the biological role of biomaterials in what is essentially biomaterial-assisted remodeling, may have to be reconsidered.

### Bladder Tissue Engineering

There are several serious conditions that affect the bladder, such as congenital or traumatic neurogenic bladder, bladder exstrophy, hemorrhagic cystitis and cancer, for which existing treatments are not always satisfactory and where regenerative medicine approaches appear attractive. Attempts to tissue-engineer bladder replacement or augmentation structures have been made for more than a decade. Atala et al. described the first successful procedures in 2006 (Atala et al., 2006). A Phase I study involved seven patients using autologous cells seeded into polyglycolic acid-collagen scaffolds that were wrapped in omentum to improve vascularization. As explained by Atala (2014), a Phase II study did not reproduce this success (Joseph et al., 2014). It was difficult to conclude what features of the process were significant causes of the disappointing outcome, but the editorial that accompanied this paper points to the unreasonable expectations from the scaffold materials: “*It is hard to believe that adding cells from a patient onto a scaffold will be enough to regenerate the organ without taking into account the normal physiological factors and necessary developmental cues. Although the bladder is a forgiving organ, it is complex, dynamic and normally expected to contract volitionally. These specific functional characteristics (contractile, impermeable, capacious, and compliant) should be considered when tissue engineering the bladder. Hence, the scaffolds used to tissue engineer bladders and the cells to be seeded on the scaffold need to be primed to work in harmony and with mutual reciprocity to allow the cell seeded scaffolds to maintain the resiliency and functionality of a normal bladder (contractile yet impermeable). The scaffold nature and its mechanical properties, the cells and their sources, and the in vitro factors before implantation may be critical but the in vivo post-implantation environment (prompt blood supply, altered scaffold mechanical properties, and seeded cell degradation and possible immune response) should not be underestimated*.”

Although some publications still suggest that there is good potential for tissue engineered bladders (Van Ba et al., 2015), the evidence for optimism is rather scant; some studies suggest a lack of understanding of bladder biomechanics may be a major factor (Ajalloueian et al., 2018). The poor prospects for synthetic biodegradable polymers has been emphasized (Pokrywczynska et al., 2014). Furthermore, the potential advantages for natural biopolymers (e.g., collagen-fibrin multi-layers, incorporating bioactive factors such as IGF-1), has been demonstrated (Vardar et al., 2016).

### Cardiovascular Tissue Engineering

Outcomes of regenerative medicine approaches to the cardiovascular system in general, and biomaterials-based tissue engineering techniques in particular, are difficult to assess, but it is clear that success has been elusive. In relation to therapies for the failing heart, Vunjak-Novakovic has recently reflected on the fact that the main focus of cardiac tissue engineering, the development of a standard of care based on cell therapy, is still without clinical application after two decades of experimentation (Vunjak-Novakovic, 2017). She poses the question of whether cell engraftment is necessary for heart repair and suggest the possibility of cell-free heart repair using cocktails of cell-secreted factors, mass produced in culture, and delivered in biomaterial patches: “*One can envision a new cell-free approach to sustained delivery of regulatory factors, controlled over time by the degradation and release kinetics of a collagen patch.”*

With respect to the myocardium and the treatment of myocardial infarction, it is clear that the conventional view of tissue-engineering scaffolds will not apply; far more relevant is the concept of an injectable biomaterial that delivers signals, and possibly cells. As discussed by Camci-Unal et al. (2014), it is the environmental stress associated with the lack of a three-dimensional flexible biomimetic microenvironment and the direct exposure of any cells injected into the myocardium to oxygen tension, free radicals and inflammatory cytokines, that leads to the death of most injected cells. This gives rise to a need for engineered biomaterials to efficiently deliver cells to the myocardium, and hydrogels are the most likely candidates. Both natural and synthetic hydrogels have been considered. In the latter case, poly(ethylene glycol) (PEG), poly(2-hydroxyethyl methacrylate) (PHEMA) and poly(N-isopropylacrylamide) (PNIPAAM) have some relevant properties, but without additional biological activity and functionalization are unlikely to satisfy all of the requirements. Since the cardiac environment is highly dynamic, tough elastomeric hydrogels should provide optimal compliance with the tissues and conductive materials may facilitate propagation of electrical signals during cardiac function (Camci-Unal et al., 2014).

Once again, native ECM molecules may form a better basis for the required hydrogels, with interest shown in collagen and fibrin. Since it is proving difficult to provide such hydrogels with appropriate stiffnesses, more subtle molecular engineering approaches may be necessary. Wu et al. (2017) have described the development of what they call “small molecular hydrogels” that are based on peptides such as ^D^FEFK^D^FEFKYRGD, which was shown to provide a scaffold for hepatocyte growth factor–modified mesenchymal stem cells that could preserve cardiac function after an infarction whilst alleviating ventricular remodeling in an animal model. These are, of course, very different to the historically-defined tissue engineering scaffolds, but it is in this direction we must look. There will be many other alternative, unconventional approaches, including cell sheet engineering, which has taken a long time to reach clinical trial stages after early enthusiasm (Miyagawa et al., 2017).

The area of heart valve replacement provides a different and interesting perspective on the role of tissue engineering. Generally this is not an area of un-met clinical need since there are perfectly adequate implantable devices, either surgically or catheter delivered for the vast majority of patients. The major potential for tissue-engineered valves lies in pediatric cases, where there is a need for devices that could adapt to growing children. This possibility received a significant setback nearly 20 years ago when cryopreserved decellularized porcine valves were implanted in 4 patients in a European clinical trial. Three of the patients died within a year (the valve in the fourth was explanted prophylactically just after implantation) because of severely inflamed and degenerated valve leaflets (Simon et al., 2003). Similar, although not so dramatic results were reported in 2012 when decellularized xenogeneic tissue-engineered pulmonary valve conduits were used for right ventricular outflow tract reconstruction in 93 patients (Perri et al., 2012). Histological analysis of explanted conduits showed inflammatory giant cells with poor autologous cell seeding, 35% of patients experiencing conduit failure and 29% conduit dysfunction. Whether better results will be obtained with synthetic polymer-based conduits remains to be seen; Benink et al. have reported “safety and functionality” in a sheep study of a synthetic polymer conduit made oif a hybrid structure of a polycaprolactone-based 2-ureido-4[IH] pyrimidinone and polycarbonate-based 2-ureido-4[IH] pyrimidinone (Bennink et al., 2018).

Vascular tissue engineering does represent a huge area of un-met clinical need. As discussed by Chang and Niklason (2017), the opportunities are considerable, but so are the challenges. Any tissue-engineered part of the vascular system has to withstand physiological pressures without leakage or aneurysm formation, which remains as a formidable barrier to reconstructing vessels in the major circulation. There have been a few successes but the vast majority of experience has been confined to animal studies. Most synthetic degradable polymers, including polycaprolactone and poly(lactic acid), tend to show limited cell infiltration with poor neotissue formation. Combining such polymers with natural biopolymers may give better performance, such as the PCL-chitosan combination used by Fukunishi et al. in a sheep model (Fukunishi et al., 2016) but it seems that we are a long way off providing a clinically-acceptable template material for arterial regeneration.

### Spinal Cord Injury

A final potential clinical application to be discussed, briefly, is that of spinal cord regeneration after injury (SCI). To date, therapies for SCI have largely been ineffective, primarily because of the need for mechanical support around the lesion in order to guide and support axonal regeneration, the absence of sufficient neurotrophic stimulation and growth-factor mediated neuroprotection, and the presence of various myelin or reactive glia derived inhibitors of axonal growth. This would seem to be a strong candidate for tissue engineering solutions, involving some form of template that could guide regeneration together with the delivery of appropriate cells and bioactive molecules that could stimulate the desired regenerative activity whilst inhibiting the undesirable negative effects. There have been many attempts to use synthetic biodegradable tubular conduits, for example the recently published trimethylene carbonate—caprolactone copolymer conduit that contains oriented poly-p-dioxanone microfilaments (Novikova et al., 2018), but such work is still mostly confined to small animal studies, where results may show some attractive features but tend to be inconclusive.

Once again, results with natural polymers have generally proven to be better. Dai and colleagues have worked with bovine collagen scaffolds, showing good results in a chronic SCI canine model when seeded with umbilical cord mesenchymal stem cells (Li et al., 2017), and then in human clinical trials with both acute (Xiao et al., 2018) and chronic (Xiao et al., 2016) spinal cord injuries.

## The Critique of Scaffolds and the Need for Active Templates

### Conventional Scaffolds

A recent conference on definitions in biomaterials science (Zhang and Williams, 2018) determined that a scaffold is “A biomaterial structure that serves as a substrate and guide for tissue repair and regeneration.” This represents the generally-perceived concept of tissue-engineering scaffolds that has persisted for the 30-year history of regenerative medicine. It is clear, however, that this conceptual definition does not even hint at what type of biomaterial can act in this way, let alone imply what are the broad specifications for scaffold materials, a point discussed by the present author a few years ago (Williams, 2014a). The reality is that there were two main specifications for tissue-engineering scaffolds at the beginning of this era; the first was that the material had to be degradable so that it could be replaced by the engineered issue that was forming, while the second was that this degradable material had to have had prior approval by the FDA for use in medical devices. This is why the first scaffold biomaterials were the bioabsorbable materials used in approved devices such as sutures, plates and drug delivery systems.

That tissue-engineering scaffolds could be (although not necessarily invariably) bioabsorbable is not contentious, but, by itself, is an insufficient criterion. This will be discussed a little later in this section. The requirement for prior FDA approval in medical devices is, however, not just irrelevant but dangerous. As the present author has discussed on several occasions (Williams, 2009, 2014b) regulatory approval for medical devices, which encompasses the biomaterials from which they are made, is predicated on the ability to show that the material does no harm; in regulatory and standards language, this determines that the materials are “biologically safe.” Thus, depending on the precise application, the materials are subjected to the biological safety tests of ISO 10993 (International Standards Organization, 2018) to show that they pass the tests that demonstrate a lack of cytotoxicity, acute systemic toxicity, reproductive toxicity, thrombogenicity, complement activation and so on. Most of these tests require that the material or device is incubated in a specified solution (typically saline) for a short period of time, then the resulting extraction solution that contains any substance that is leached from the material is exposed to cells in culture, or to an appropriate *in vitro* or *in vivo* test system, and the results compared to actual or historic controls. Any hint that the extract produces a greater response compared to a control is interpreted as a failure, such that the material or device is considered biologically unsafe, and unsuitable for implantation in humans.

For biomaterials in medical devices, this requirement for minimal biological activity has resulted in the restriction of acceptable materials to those that have maximum chemical and biological inertness; that is the reality, inertness wins. Whenever the choice has deviated from inertness, problems have usually been encountered. There is some suggestion with implantable devices that inert materials themselves do not necessarily produce optimal results in all situations. For example, vascular grafts need help from endothelial cells to generate a superior neointima (Zilla et al., 2007) intravascular stents require help from anti-proliferative drugs to control in-stent re-stenosis (Otsuka et al., 2012), spinal fusion devices may give better outcomes when assisted by locally-released bone morphogenetic proteins (Axelrad and Einhorn, 2009) and thrombosis of heart valves is deterred by systemic anticoagulants (VanderLaan et al., 2012). A detailed analysis of the biocompatibility of these materials and applications (Williams, 2017b) show that, at maximal inertness, the host response is controlled by biomechanical factors, the release of particulate matter (e.g., wear particles in joint replacements) and applied pharmacological factors.

If biomaterials are used in applications other than implantable devices, where mechanical and physical functionality dominate the specifications, for example in drug and gene delivery processes, imaging and diagnostic systems and regenerative medicine applications, quite different requirements apply. This is why conventional tissue-engineering scaffolds, based upon FDA predicate considerations, are unlikely to result in new tissue generation; the materials used have to do more than guide tissue regeneration.

### Tissue Engineering Templates

The above arguments make it clear that the originally conceived requirements for scaffold materials were, at best, too simple and, at worst, very misleading. Several of the early tissue-engineering biomaterials were modeled on those absorbable materials that had been used in FDA approved surgical sutures, e.g., synthetic polymers based on the aliphatic polyesters such as poly(glycolic acid). It is true that some of these polymers could degrade in the body without any clinically relevant adverse responses. However, a surgical suture was not designed to take part, biologically, in wound healing; it was required to hold tissues together while the repair process tool place according to natural healing mechanisms, and then degrade and resorb with minimal host response. Nothing could be further from the main requirement of a tissue-engineering biomaterial, which should be to actively take part in the process of tissue regeneration. No wonder that in those clinical applications described in section Introduction above, the simple concept of using an FDA approved synthetic degradable polyester rarely worked, and that the more the biomaterial choice moved toward natural or naturally-derived substances, the chances of regenerating tissues improved.

This, of course, is not the full story; a scaffold made from collagen, fibrin or silk may have a better chance, but far more attention has to be paid to the morphology and architecture of the construct, since the ability to signal to the target cells is not solely based on chemistry. The more conventional polymeric (or even resorbable ceramics such as some calcium phosphates) scaffolds were usually made by solid free form fabrication techniques or electrospinning. The microscale porous structures had various morphological characteristics, often with a degree of anisotropy, but their design rarely attempted to replicate a biological microenvironment. The question then arises as to whether these structures specifically replicate the niche of the target cells, i.e., those that are intended to be the focus of tissue regeneration. Moreover, the niche of these target cells, often but not necessarily stem cells, changes with time during the process of extracellular matrix expression; will electrospun collagen fibers change in the same way in order to accommodate this natural process? Even, therefore, with biomaterials that have some latent or intrinsic biological activity that could potentially be harnessed for stem cell signaling, it is unlikely that such activity can be sustained during the tissue regeneration process.

This is why the simple concept of a scaffold guiding a tissue regeneration process is insufficiently robust to result in effective, sustainable, tissue engineering. This is also the reason why the concept of a template is preferred to a scaffold. In general usage, a scaffold is a structure that mechanically facilitates the building of a construct, which is then disassembled and removed at the end of a process, without actually playing any part of that building process. In tissue engineering, the biomaterial has to do much more than that; for example a tissue-engineering template could be defined as “a biomaterials-based structure of defined size, chemistry and architecture that controls the delivery of molecular and mechanical signals to target cells in tissue engineering processes.”

It is, perhaps, surprising that the tissue-engineering field as progressed up to this point without any authoritative presentation of specifications for the biomaterials. The present author provided a reasonably comprehensive list of such specifications, some mandatory and some optional depending on the application (Williams, 2014a). The more important of these template specifications may be summarized as follows:

- The template should recapitulate the architecture of the target cells,
- The template should be capable of adapting to the constantly changing microenvironment,
- The biomaterial should be degradable if that is desired, with appropriate degradation kinetics and appropriate morphological and chemical degradation profiles,
- The biomaterial should be capable of orchestrating molecular signaling to the target cells, either by directing endogenous molecules or delivering exogenous molecules,
- The biomaterial should have appropriate elastic/viscoelastic properties that favor mechanical signaling to the target cells,
- The biomaterial should be injectable if that is desired, with appropriate rheological characteristics, and transformation mechanisms and kinetics,
- The biomaterial should be capable of forming into an architecture that optimizes cell, nutrient, gas and biological molecule transport, either *ex vivo, in vi*vo or both, and which facilitates blood vessel and nerve development,
- Where necessary, the biomaterial should be compatible with the processing techniques that simultaneously pattern both the material and the cells; this has become an important issue in the context of 3D bioprinting, with features such as viscosity having a significant role,
- The material should be intrinsically non-cytotoxic, non-immunogenic and minimally pro-inflammatory.

On the basis of these quite specific requirements, a series of essential characteristics emerge (see Table 1). In order to recapitulate cell microenvironments, a substance that resembles the architecture and biochemical features of the desired, but acellular, new tissue seems an obvious choice The use of decellularized tissue, which represents the complex extracellular matrix (ECM) environment, has become a popular choice, both experimentally and clinically, for these templates (Urciuolo and De Coppi, 2018). A very significant point here is that the ECM is not simply a collection of proteins, or even a three-dimensionally arranged collection of proteins. The ECM components interact with each other in specific ways; these interactions between components, and between different isoforms of the same component, are not only tissue-specific but site-specific within each defined tissue. Such interactions are unlikely to be efficiently achieved by chemical or processing manipulations of collections of ECM components, even if some micro- and nano-architectural features can be replicated. On the contrary, this is most likely achievable when the complete structural features of the normal ECM can be prepared, which can occur if natural tissues, most likely of xenogeneic origin, can be decellularized.

**Table 1:** Comparison of synthetic polymers, biopolymers and ecm-derived materials for tissue engineering templates.

**Synthetic polymers**	**Biopolymers**	**ECM-derived materials**
Good control of essential material chemical characteristics (mol.wt etc.), giving acceptable quality control and regulatory processes	Material characteristics depend on source and processing conditions	Considerable variability in essential material characteristics, making quality control difficult
Usually very cost effective	Can be very expensive, especially if recombinant techniques are required	Cost effectiveness will vary with source and processing conditions
Mechanical properties usually tunable and cover wide range	Mechanical properties vary, some can be quite good, others poor	Mechanical properties vary, some can be quite good, others poor
Degradation characteristics can be tunable and cover wide range	Degradation characteristics can be tuned, especially by control of blends	Degradation properties not easily tuned
Materials are inherently incompatible with support of key cell functions; may be capable of functionalization for some limited improvement	Careful choice of formulation can give good compatibility with cell function	Structure most closely replicates normal cell microenvironment, with support of cell function
Most materials should be free from toxicological and immunological risks	Most, although not all, materials should be free from toxicological and immunological risks	Possibility of immunological responses
Only a few are compatible with 3D bioprinting	Several materials with excellent characteristics for bioprinting	Not ideal for bioprinting

As discussed recently by Hussey et al. (2018), ECM based materials can be prepared in several ways. Tissue decellularization may involve tissues such as the small intestine, the urinary bladder or the dermis, which can be subjected to mechanical delamination and immersion in decellularization agents, yielding a 2D sheet or a hydrogel. Several commercial products have been approved by the FDA for use in soft tissue repair, including ventral hernia repair and breast reconstruction. The clinical outcomes are controlled by the surgical technique, the matching of the sourced material with the specific clinical conditions, the age of the sourced material and patient comorbidities. Mechanistically these outcomes are determined by the host response to the ECM derived material, including angiogenesis, innervation, stem cell recruitment and modulation of the immune response. Although it is often claimed that this decellularized tissue does not elicit adverse innate or adaptive immune responses, this may not be the case. For example, although the ECM product CorMatrix® continues to receive regulatory approval in several jurisdictions, a systematic review of cardiovascular applications for this porcine SIS material suggests that the long-term histopathological data indicates the presence of significant chronic inflammation; a dense eosinophilic inflammation with granulation tissue and fibrosis and without tissue remodeling is not consistent with the required biocompatibility (Mosala Nezhad et al., 2016).

An alternative to tissue decellularization is whole organ decellularization. This usually involves perfusion of the organ, for example heart, liver, lung or kidney, through the native vasculature, followed by recellularizaton using patient-derived cells and subsequent transplantation into the recipient. This, of course, is not a trivial process and, after a decade or more, progress toward clinical transplantation has been slow. With respect to the kidney, for example, Petrosyan et al. have described the challenges with the repopulation of the renal matrix with functional renal cell types (Petrosyan et al., 2016). Zhou et al. have reported on progress with porcine decellularized lung scaffolds seeded with human airway epithelial progenitor cells derived from rejected donor lungs and banked human umbilical vein endothelial cells (Zhou et al., 2018). When transplanted into porcine recipients, the grafts were able to withstand the recipient's pulmonary circulation and exchange gases during ventilation over a short period of time. This demonstrates the feasibility of this approach to lung tissue-engineering templates, but there is still a long way to go before full graft maturation can be achieved.

It should be mentioned in passing that one theoretical alternative to the decellularization and recellularization of animal-derived organs is the use of direct xenotransplantation. It could be argued that this would not constitute a tissue engineering approach, or possibly even a regenerative medicine approach but it should be considered here. Although xenotransplantation has been suggested, and experimented, for many decades, going back to the attempts to transplant baboon hearts into pediatric human patients in the 1980s (Bailey et al., 1985), the pathobiologic barriers, especially those of immunological and infectivity character, not to mention the ethical implications, have appeared insurmountable. Recently, however, with the advances in genetically engineered pigs and better immunosuppressive agents, the reality of xenotransplantation as a solution to organ donor shortage is becoming closer (Cooper et al., 2018), possibly rendering whole organ decellularization less relevant.

As a final point with respect to templates, several of the examples of improved success discussed in section Introduction referred to biomaterials, either synthetic or natural, which did have greater biological activity and whose selection was not predicated on inertness. Usually these may be considered as hydrogels (Williams, 2018) or elastomers (Coenen et al., 2018). Virtually all reviews and analyses of these materials point to the limited biocompatibility (with respect to tissue engineering) of synthetic versions and the limited mechanical functionality and irreproducibility of many natural versions. With synthetic materials, progress is undoubtedly being made, as, for example, with click chemistry approaches to polymer synthesis that can eliminate all toxic by-products of polymerization (Xu and Bratlie, 2018) but it does seem inappropriate that so much work is being performed on variations of these synthetic polymers, and thousands of papers on them published each year, when they are based on the wrong principles of tissue-engineering templates. With natural materials, even with some continuing disadvantages, there is far more hope that the correct principles can guide their development. It does seem likely that some single-protein based materials (such as tropoelastin or collagen) or some protein-protein hybrids, especially if they can be appropriately functionalized and prepared with optimal architecture, provide a better pathway to those template specifications.

## Conclusions

This essay has shown, through a series of examples of attempts to regenerate tissues through a conventional scaffold approach, that any process that relies on biomaterials that have been determined to be biologically inert and, therefore “biologically safe” is highly unlikely to succeed. It is possible that some new tissue will form within the porosity of these scaffolds, but that is usually in spite of rather than because of their properties. It is stated clearly and unequivocally here that the conventional scaffold paradigm, especially when based on synthetic biodegradable polymers, is mechanistically inappropriate and that a far better paradigm is one that is based on tissue engineering templates which, far from being inert, actively take part, both biologically and mechanically in the stimulation of those target cells intended to regenerate new tissue. It is further believed that although some natural biopolymers, as hydrogels or elastomers, and especially when used in conjunction with the right signaling molecules, can produce some good results experimentally, these, in their present form, are not the basis for sustainable tissue engineering. The most attractive biomaterials from the tissue engineering perspective are those derived from decellularized tissues, although even here biocompatibility characteristics are not fully understood and sustainability is still some way off.

## Data Availability

All datasets analyzed for this study are included in the manuscript and the supplementary files.

## Author Contributions

The author confirms being the sole contributor of this work and has approved it for publication.

### Conflict of Interest Statement

The author declares that the research was conducted in the absence of any commercial or financial relationships that could be construed as a potential conflict of interest.
